# LOOKING FORWARD TO A NEW DECADE OF TRANSLATING DATA INTO TOOLS TO SUPPORT STATE LEADERS ADVANCING HEALTH EQUITY

**DOI:** 10.1093/geroni/igae098.0301

**Published:** 2024-12-31

**Authors:** Elizabeth Dugan

**Affiliations:** University of Massachusetts Boston, Boston, Massachusetts, United States

## Abstract

This presentation will describe how the healthy aging data reports started and how the work has evolved. The importance of community-engagement, collaboration, and shared ownership has driven progress. The partnership of the Point32Health Foundation has been critical to our growth and success. Opening ways for other funders to support the work has allowed us to partner with other states to expand the reach and impact of the reports. How graduate gerontology students have contributed innovations and improvements to our process and products is highlighted.

